# The Prognostic Significance of MACC1 Expression in Breast Cancer and Its Relationship to Immune Cells in the Tumor Microenvironment and Patient Survival

**DOI:** 10.3390/medicina57090934

**Published:** 2021-09-05

**Authors:** Dina A. Ali, Dina M. El-Guindy, Mohamed A. Elrashidy, Nesreen M. Sabry, Ahmed M. Kabel, Rasha A. Gaber, Rowida R. Ibrahim, Sara M. Samy, Marwa M. Shalaby, Samir A. Salama, Dina Abdelhai

**Affiliations:** 1 Clinical Pathology Department, Faculty of Medicine, Tanta University, Tanta 31527, Egypt; drdinaadam012@gmail.com (D.A.A.); dinaibraheem85@yahoo.com (D.A.); 2 Pathology Department, Faculty of Medicine, Tanta University, Tanta 31527, Egypt; dina-elguindy@hotmail.com (D.M.E.-G.); mohamedrashidy@yahoo.com (M.A.E.); 3 Clinical Oncology Department, Faculty of Medicine, Tanta University, Tanta 31527, Egypt; nesreensabry1eg@yahoo.com; 4 Pharmacology Department, Faculty of Medicine, Tanta University, Tanta 31527, Egypt; 5 Medical Biochemistry Department, Faculty of Medicine, Tanta University, Tanta 31527, Egypt; dr_rashagaber@yahoo.com (R.A.G.); rowaida.yousef@med.tanta.edu.eg (R.R.I.); 6 Medical Microbiology and Immunology Department, Faculty of Medicine, Tanta University, Tanta 31527, Egypt; dr.sms2006@hotmail.com (S.M.S.); Dr_memo_2004@yahoo.com (M.M.S.); 7 Division of Biochemistry, Department of Pharmacology, College of Pharmacy, Taif University, P.O. Box 11099, Taif 21944, Saudi Arabia; s.salama@tu.edu.sa

**Keywords:** metastasis-associated colon cancer-1, breast cancer, CD163+ tumor-associated macrophages, CD56+ natural killer cells, CD8+ cytotoxic T lymphocytes

## Abstract

Breast cancer (BC) is one of the most prevalent malignancies among females worldwide. Globally, distant metastases were reported to be responsible for a large proportion of breast cancer-related deaths. The metastasis-associated colon cancer-1 (MACC1) gene was reported as a reliable biomarker for early detection of metastasis and prediction of prognosis of breast cancer. This study investigated the prognostic significance of MACC1 in breast cancer in relation to the clinicopathologic characteristics and patients’ survival. Furthermore, the possible correlation between MACC1 expression and the different immune cells in the tumor microenvironment was explored. MACC1 mRNA was identified using quantitative reverse transcription polymerase chain reaction in 120 breast cancer specimens and adjacent non-cancerous tissues. MACC1 mRNA expression was significantly higher in the cancerous relative to the non-cancerous tissues (*p* < 0.001). High MACC1 expression was significantly associated with poor prognostic parameters, such as larger tumor size, grade III tumors, positive nodal metastasis, lymphovascular invasion, stage III tumors, and elevated *Ki-67* expression. Higher MACC1 mRNA levels were positively correlated with CD163+ tumor-associated macrophages (r = 0.614, *p* < 0.001), and were negatively correlated with CD56+ natural killer cells (r = −0.398, *p* < 0.001) and CD8+ cytotoxic T lymphocytes (r = −0.323, *p* < 0.001). MACC1 expression was associated with poor patient overall survival (OS) and progression-free survival (PFS) (*p* < 0.001). Multivariate analysis suggested that MACC1 expression and the presence of lymphovascular invasion could be independent prognostic indicators for breast cancer (*p* = 0.015 and 0.042, respectively). In conclusion, MACC1 is highly expressed in cancerous tissues and is significantly related to poor prognostic factors, overall survival, and progression-free survival. MACC1 may influence infiltration of the immune cells in the tumor microenvironment, enhance immune escape of tumor cells, and may serve as a reliable independent prognostic factor for breast cancer.

## 1. Introduction

Breast cancer (BC) is the most frequently occurring malignancy in women and is the second most prominent cause of cancer-associated death in women all over the world [1]. Ongoing BC treatment options consistently encompass surgery if the disease is diagnosed early. Depending on the stage and the molecular subtype when diagnosed, BC treatment may involve hormonal therapy, chemotherapy, and/or radiation. For years, BC was not considered a highly immunogenic tumor type, particularly in comparison with melanoma or lung cancer. However, recent studies reported significant immune cell infiltration consisting of both innate and adaptive immune cells within BC tumor microenvironment [2].

Metastasis-associated colon cancer gene-1 (MACC1) was first described as a c-MET transcription regulator that mediates cancer colon progression and metastasis by its ligand hepatocyte growth factor (HGF). MACC1 was overexpressed in primary and metastatic cancer colon and was associated with poor prognosis [3]. High expression of MACC1 was also proven to be related to poor clinical outcomes in a wide variety of tumors, such as gastric, endometrial, and ovarian carcinomas [4,5,6].

Besides its role in tumor development and metastasis, it was believed that MACC1 protects metastasis from immune destruction by modifying the tumor microenvironment (TME) [7]. TME consists of inflammatory and immune cells, in addition to fibroblasts, blood vessels, and the extracellular matrix [8]. There is a growing number of studies indicating that TME, particularly tumor-infiltrating immune cells (TIICs), exerts an essential function in tumor development and is largely related to patients’ prognosis [9].

Tumor-associated macrophages (TAMs) represent an important constituent of TME. TAMs could differentiate into either M1- or M2-polarized macrophages, which have opposing functions on tumors. Activated M1 macrophages have cytotoxic effects on tumor cells. They secrete pro-inflammatory cytokines and activate type 1 T-cell mediated immune responses. On the other hand, M2 macrophages promote tumor growth, angiogenesis, and suppress adaptive immunity [10]. CD163 is a highly specific monocyte/macrophage marker for polarized M2 macrophages. High CD163+ TAMs density was proven to be associated with poor prognostic parameters in breast cancer [11].

CD56 is a neural cell adhesion molecule that is commonly expressed on natural killer (NK) cells. NK cells are unique as they participate in both innate and adaptive immune mechanisms [12]. They produce various pro-inflammatory cytokines, which stimulate propagation of other immune cells. In addition, they may directly kill the tumor cells themselves without prior sensitization [13]. However, the competence of NK cells to identify and destroy tumor cells is decreased in cancer. In BC patients, decreased NK cell infiltration correlates with tumor progression and invasiveness [14].

CD8 molecules serve co-receptors for major histocompatibility (MHC) class I restricted antigen recognition. CD8 is extensively used as a marker of cytotoxic T-lymphocytes. CD8+ cytotoxic T-lymphocytes (CTLs) comprise the dominant elements of tumor-infiltrating lymphocytes. These cells are pivotal components of the tumor-specific immunity that attacks malignant cells. Activated CD8+ CTLs play fundamental cytotoxic functions, including degranulation and elaboration of IFN-γ, which could induce tumor apoptosis and inhibit angiogenesis [15]. A high density of CD8+ CTLs has been linked with favorable clinical outcomes in various malignancies including BC [16].

The significance of immune cells in BC has been largely investigated. However, the relation between MACC1 and tumor-infiltrating immune cells is still unclear. In the present work, we aimed to evaluate the expression of MACC1 in BC and its relation to the clinicopathological aspects and patients’ survival. The current study was extended to investigate the correlation between MACC1 expression and CD163+ tumor-associated macrophages, CD56+ natural killer cells, and CD8+ cytotoxic T lymphocytes within BC tumor microenvironment.

## 2. Subjects and Methods

### 2.1. Study Population and Design

This prospective study was conducted in the Clinical Pathology, Pathology and Clinical Oncology Departments, Faculty of Medicine, Tanta University, Egypt. The study included 120 patients with primary breast cancers operated upon by modified radical mastectomy during the period between January 2016 and December 2017. Patients were followed up for 3 years till December 2020. The study protocol was approved by the institutional ethics committee following the Declaration of Helsinki and a signed informed consent was obtained from all included patients before data collection.

Patients eligible for this study included all patients with pathologically confirmed primary BC and complete clinical and outcome data. Cases with recurrent BC, benign breast conditions, bilateral breast cancer, and those with other malignant conditions were excluded.

### 2.2. Breast Tissue Specimen

Breast cancer tissues obtained by modified radical mastectomy were taken to the Pathology Department for histopathological examination and immunohistochemical (IHC) staining. Samples were taken from the tumor mass and the adjacent non-cancerous tissues and were directly stored at −80 °C for molecular study.

### 2.3. Histopathological Evaluation

After fixation of BC specimens in 10% neutral buffered formalin, paraffin blocks were prepared. Staining with hematoxylin and eosin (H&E) was carried out to confirm the diagnosis of BC. All included cases were categorized as invasive carcinoma of no special type according to the World Health Organization (WHO) criteria [17]. The tumor grade was determined according to the Nottingham grading system [18]. TNM staging was applied according to the American Joint Committee on Cancer [19]. Evaluation of standard biomarkers expression, including estrogen receptors (ER), progesterone receptors (PR), HER2 (human epidermal growth factor receptor 2), and Ki-67 was routinely performed.

### 2.4. Detection of MACC1 Gene Expression by Quantitative Reverse Transcription PCR

RNA extraction kits (RNeasy Mini Kit, Qiagen, Hilden, Germany, CAT # 74104) were used to extract total RNA from each frozen breast tissue sample according to the manufacturer’s instructions. Concentrations were measured quantitatively at 260 nm absorbance using Jenway UV/visible spectrophotometer 6305, Staffordshire, UK. QuantiTect^®^ Reverse Transcription (Qiagen, Hilden, Germany, CAT # 205311) was used to reverse transcribe the extracted RNA. TaqMan Gene Expression Assay kits (Thermo Scientific, Waltham, MA, USA) were used for RT-PCR amplification with relative quantification of MACC1 mRNA expression.

The primers’ sequence for MACC1 was as follows: forward 5′-GACCAGGCAATCATTACGGC-3′ and reverse: 5′-CCCAGCAGTCTGTTTCACCAAG-3′ [20]. Control GAPDH assay was used for each sample (forward primer: 5-ACCACAGTCCATGCCATCCAC-3; reverse primer: 5-TCCACCACCCTGTTGCTGTA-3). The plate was applied on real-time PCR system (Applied Biosystems, step I version) in the following thermal profile: hold at 95 °C for 10 s followed by 40 cycles (denaturation at 95 °C for 15 s and annealing/extension at 60 °C for 20 s). The cycle threshold (CT) was obtained for the gene using Applied Biosystems, step I version, software analysis modules, and the expression of the gene was relatively quantified using the equation 2^−ΔΔCT^ [21].

### 2.5. Immunohistochemical Staining

Sections form BC specimens (5 μm thick) were prepared on positively charged slides and left to dry for 30 min at 37 °C. Deparaffinization and antigen retrieval were accomplished using Dako PT Link unit. High pH EnVision^TM^ FLEX Target Retrieval Solution was used going up to 97 °C for 20 min. Dako Autostainer Link 48 was applied for immunostaining. Antibodies included in this study were CD163 rabbit monoclonal antibody (clone EP324, Medaysis, CA, USA), CD56 mouse monoclonal antibody (Clone 123C3, ready-to-use, Dako-Agilent, Glostrup, Denmark), and CD8 mouse monoclonal antibody (Clone C8/144B, 1:300 dilution, Dako-Agilent, Glostrup, Denmark). Briefly, slides were kept in peroxidase-blocking reagent for 5 min, incubated with primary antibodies for 20–30 min, horseradish peroxidase (HRP) polymer reagent for 20 min, and diaminobenzidine (DAB) chromogen/substrate working solution for 10 min. Lastly, slides were counterstained using hematoxylin.

#### Quantification of Tumor-Infiltrating Immune Cells

CD163, CD56 and CD8 stained slides were assessed by two pathologists, blinded to the clinical background, to determine areas with maximum TAMs, NK cells and CD8+ CTLs infiltration. Sections were evaluated at a distance away from areas of necrosis. For each slide, three hotspots at ×400 magnification were decided for immune cells counting. The total number of the stained cells, regardless of the intensity, were counted manually using the plug-in “cell counter” in the Image J software (Java image processing program inspired by National Institutes of Health, Bethesda, MD, USA). The mean of the three counts was calculated for each case [22,23].

### 2.6. Treatment and Follow-Up

Following surgery, patients received their adjuvant therapy in the form of chemotherapy, radiotherapy, hormonal, and targeted therapies. Treatment was determined according to the treatment protocols of the Clinical Oncology Departments, Tanta University, Egypt. Chemotherapy was either 4 cycles AC (anthracycline, cyclophosphamide) alone, or 4 cycles AC followed by 4 cycles of paclitaxel, according to tumor size, Ki-67 status, and nodal involvement. This was followed by adjuvant radiotherapy.

Cases positive for hormone receptors received adjuvant hormonal therapy in the form of tamoxifen with luteinizing-hormone-releasing hormone (LHRH) agonists for premenopausal or aromatase inhibitors for postmenopausal females planned for 5 years. HERs2 positive cases received trastuzumab as a targeted therapy.

All patients were followed up every 3 months through clinical, radiological, and laboratory assessment. The patients who progressed received second line treatment according to the site and the type of progression.

The study end points were loco-regional recurrence, distant metastasis, death, or completing 3 years follow-up.

### 2.7. Statistical Analysis

Statistical analysis was performed using Statistical Package for Social Science (SPSS) version 23. Data were presented as frequencies for categorical variables and mean ± SD or median and range for numerical variables. The Kolmogorov–Smirnov test was used to verify the normality of distribution of variables. Differences between numerical variables were analyzed using the Mann–Whitney test for two groups and Kruskal–Wallis test for more than two groups. Correlations between MACC1 mRNA expression levels and CD163+ TAMs, CD56+ NK cells, and CD8+ CTLs counts were evaluated using the Pearson correlation coefficient.

For survival analysis, the overall survival (OS) was calculated from the date of diagnosis to the time of the last follow-up visit or death. Progression-free survival (PFS) was the time elapsed from the date of diagnosis to the date of the first evidence of disease progression or death in absence of disease progression. Kaplan–Meier test was used to construct survival curves, and to assess the significance of differences between the studied groups. The exact log-rank test was performed. Cox proportional hazard regression models were used to evaluate the prognostic factors of OS. MACC1 mRNA expression levels, CD163+ TAMs, CD56+ NK cells, and CD8+ CTL were grouped into high and low groups considering their medians as cut-off points. *p*-values <0.05 were considered statistically significant.

## 3. Results

### 3.1. The Clinicopathological Data

This study was carried out on 120 BC patients. The mean age of the studied cases was 47.3 ± 5 years. In half of the cases, the tumor size measured from more than 2 cm to less than or equal to 5 cm. Grade II constituted most of cases (73.3%) and nearly half of cases (53%) were stage III. Positive nodal metastasis was detected in 104 (86.7%) cases, whereas lymphovascular invasion (LVI) was identified in 17 (14.2%) cases.

As regards hormonal receptors’ status, seventy-two (60%) cases were positive for ER expression, whereas PR positive cases constituted 64 (53.3%) cases. HER2 expression was negative in 78 (65%) cases and Ki-67 expression was ≥15% in 70 (58.3%) cases. The clinicopathological data are summarized in Table 1.

### 3.2. Evaluation of MACC1 mRNA Relative Expression by qRT-PCR

**Figure 1:**
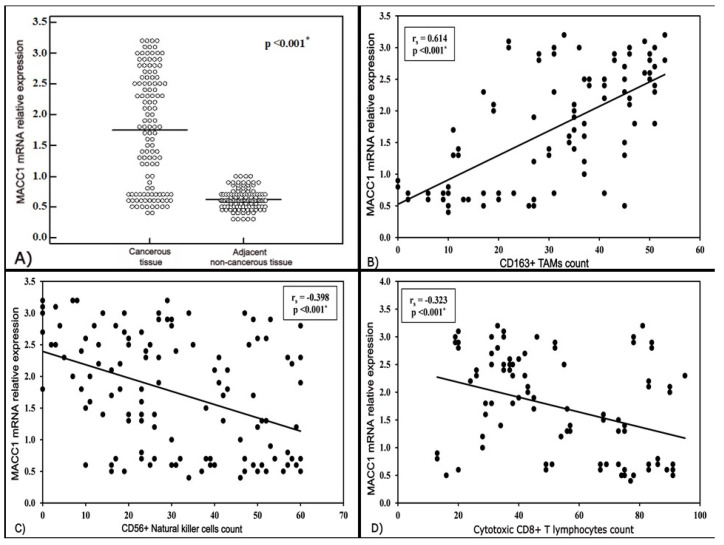
MACC1 mRNA relative expression: (**A**) Significant difference between breast cancerous and non-cancerous tissues (*p* < 0.001); (**B**) Significant positive correlation with CD163+ tumor-associated macrophages (TAMs) count (r = 0.614, *p* < 0.001); (**C**) Significant negative correlation with CD56+ natural killer (NK) cells count (r = −0.398, *p* < 0.001); (**D**) Significant negative correlation with CD8+ cytotoxic T lymphocytes (CTLs) count (r= −0.323, *p* < 0.001).

Both BC and adjacent non-cancerous tissues displayed MACC1 mRNA relative expression in all included patients. However, there was a statistically significant difference in MACC1 mRNA relative expression between the cancerous and the adjacent non-cancerous tissues (*p* < 0.001). MACC1 mRNA relative expression levels were higher in BC tissues (median 1.8, range 0.4–3.2) in relation to the adjacent non-cancerous tissues (median 0.6, range 0.3–1.0) as illustrated in Figure 1A.

### 3.3. Relation between MACC1 mRNA Relative Expression and Clinicopathological Data

Higher MACC1 mRNA relative expression levels were significantly related to larger tumors >5 cm (median 2.5, range 1.3–3.2), grade III cases (median 2.3, range 1.2–3.2), positive nodal metastasis (median 2.0, range 0.4–3.2), presence of LVI (median 2.5, range 1.6–3.2), stage III tumors (median 2.3, range 0.5–3.2), and high Ki-67 ≥15 (median 2.3, range 0.5–3.2) (*p* < 0.001 for all). Among the different molecular subtypes, MACC1 mRNA relative expression levels differed significantly (*p* < 0.001), whereas no significant associations were detected between MACC1 mRNA relative expression and the patients’ age, menopausal status, ER, PR, and HER2 expression, as summarized in Table 2.

### 3.4. Immune Cells (CD163+ TAMs, CD8+ CTLs, and CD56+ NK Cells) in TME and Their Correlation with MACC1 mRNA Relative Expression

**Figure 2:**
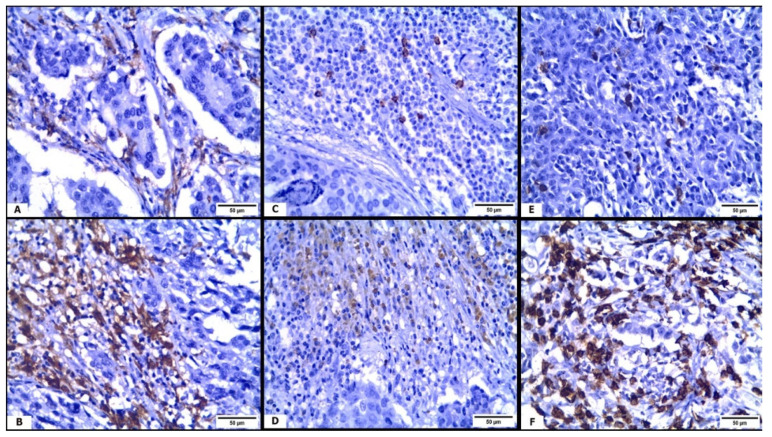
Immune cells in breast cancer tumor microenvironment (×400): (**A**) CD163 immunohistochemical (IHC) expression highlights few tumor-associated macrophages (TAMs) within tumor stroma; (**B**) CD163 IHC stain indicates dense stromal TAMs infiltration; (**C**) CD56 IHC stain demonstrates few scattered natural killer (NK) cells within tumor stroma; (**D**) CD56 IHC expression highlights large number of stromal NK cells; (**E**) CD8 IHC stain demonstrates few intra-tumoral cytotoxic T lymphocytes (CTLs); (**F**) CD8 IHC expression highlights abundant intra-tumoral CTLs.

The mean CD163+ TAMs count in the studied cases was 31.6 ± 14.8. The mean CD56+ NK cells count was 30.8 ± 18.1, whereas the mean count for CD8+ CTLs was 52.2 ± 23.4. Representative images for immune cells infiltration in BC TME are shown in Figure 2. Analyzing the correlation between MACC1 mRNA relative expression and CD163+ TAMs revealed significant positive correlation, as higher levels of MACC1 mRNA relative expression were associated with an increase in TAMs infiltration (r = 00.614, *p* < 0.001). Conversely, significant negative correlation was detected between MACC1 mRNA relative expression and CD56+ NK cells, as an increase in MACC1 mRNA relative expression was associated with a decrease in CD56+ NK cells infiltration (r = −0.398, *p* < 0.001). Similarly, the correlation between MACC1 relative mRNA expression and CD8+ CTLs exhibited significant negative correlation (r= −0.323, *p* < 0.001), as demonstrated in Figure 1B–D.

### 3.5. Relation between MACC1 mRNA Relative Expression and the Patients’ Survival

**Figure 3:**
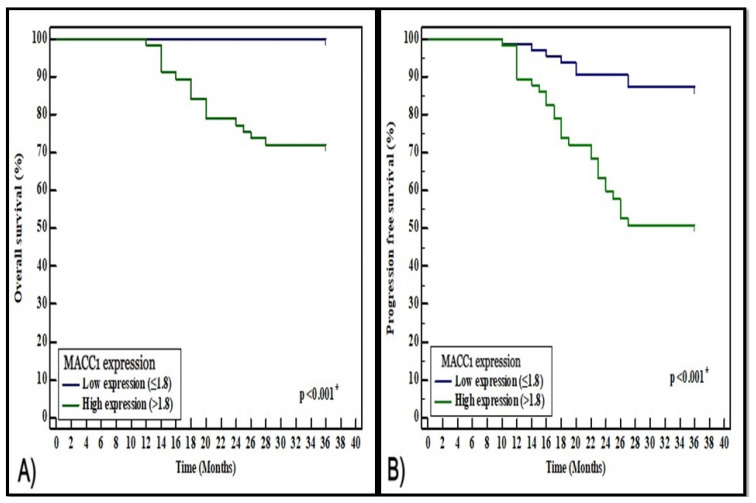
Kaplan–Meier curves for (**A**) overall survival (OS), and (**B**) progression-free survival (PFS) (**B**) of breast cancer patients with low and high MACC1 mRNA relative expression.

Patients were categorized into low and high expression groups using the median of MACC1 mRNA relative expression (1.8) as a cut-off point. Kaplan–Meier survival curve analysis demonstrated significant associations between MACC1 relative mRNA expression and both OS and PFS (*p* < 0.001 for both). The 3 years OS rates were 100% for patients with low MACC1 expression and 71.9% for patients with high MACC1 expression. Regarding PFS, the rates for 3 years PFS were 87.3% and 50.9% for MACC1 low and high expression patients, respectively, as shown in Figure 3.

### 3.6. MACC1 mRNA Relative Expression as an Independent Prognostic Factors in BC Patients

To evaluate whether MACC1 mRNA relative expression could serve clinically as an independent prognostic factor in BC, Cox proportional hazard regression models were performed to analyze the independent prognostic factors related to patients’ OS. In univariate analysis, the factors with statistical significance were LVI, tumor stage, PR, CD163+ TAMs count, CD56+ NK cells count, and MACC1 mRNA relative expression. In multivariate analysis, both LVI and MACC1 mRNA relative expression maintained their statistical significance, as demonstrated in Table 3.

## 4. Discussion

MACC1 is considered as a main regulator of tumorigenesis and cancer metastasis in primary and metastatic cancer of the colon [24]. MACC1 overexpression has also been linked with unfavorable prognosis in different tumors, such as lung cancer, gastric cancer, ovarian cancer, endometrial cancer, cervical cancer, osteosarcoma, and renal cell carcinoma [25]. It is speculated that MACC1 not only induces metastatic spread but might also save metastases from being destroyed by the immune cells and is thereby associated with poor prognosis [7].

Recently, immune evasion has been found to be a hallmark of BC. Different cells located within TME could promote tumor progression and immune evasion [26]. Tumors develop distinct mechanisms to evade immune surveillance, induce tolerance, and survive in the host [27].

Nevertheless, it is not stated whether MACC1 controls the immunological characteristics of cancer cells while enhancing the metastatic ability of the tumor cells. This study aimed to evaluate MACC1 mRNA expression in BC and its relation to the clinicopathological parameters and patients’ survival. Furthermore, the current work focused on the correlation between MACC1 mRNA expression and the different immune cells (CD163+ TAMs, CD56+ NK cells, and CD8+ CTLs) in the TME of BC.

In the present study, MACC1 mRNA relative expression levels were higher in BC tissues compared to the adjacent non-cancerous tissues. This was in agreement with Han et al. [28], who reported that MACC1 expression in the tumor tissues of triple negative BC cases was higher than in the control. Similarly, Tan et al. [29] studied the serum level of MACC1 by enzyme-linked immunosorbent assay (ELISA) and reported that MACC1 serum levels were higher in BC patients than the control and, consequently, it had a poor prognostic value. This was also confirmed by Ahmed et al. [30].

The present study detected significant association between MACC1 mRNA relative expression and poor clinicopathologic parameters of BC including larger tumors, grade III tumors, positive nodal metastasis, LVI, stage III tumors, and high Ki-67 expression. These results are in line with Tan et al. [29] who revealed that MACC1 levels in the serum of BC patients were associated with staging, nodal metastasis, tumor size, lymph nodes status, and Ki-67 status. They hypothesized that serum MACC1 levels may indicate BC progression and invasiveness. In addition, Huang et al. [31] and Dai et al. [32] showed a significant association between elevated MACC1 in the tumor tissues and poor prognostic factors, such as TNM stage, tumor size, and lymph nodes status.

Among the molecular subtypes, MACC1 mRNA relative expression was significantly different. Regarding the association between MACC1 mRNA relative expression and the hormonal receptors and HER2 status, no significant relations were observed with ER, PR, and HER2 status, which agreed with previous studies that reported the same findings [29,30,31,33].

In order to elucidate the exact molecular pathways of MACC1, various studies have demonstrated that MACC1 functions as a transcription activator for c-MET, and is important in controlling HGF-c-MET signaling pathways in tumors, which thereby enhance tumor growth, invasion, and HGF-induced scattering of the tumor cells [3]. In addition, c-MET oncogene, which codes for the tyrosine kinase receptor of HGF, could promote invasion and the metastatic potential of different cancers, including BC, through promoting angiogenesis, proliferation, invasiveness, and survival of the tumor cells [34]. In addition, c-MET overexpression in BC was linked to the disease progression and was related to poor outcomes [35].

The prognostic importance of the immune cells in BC was previously evaluated. Various studies assessed CD163+ TAMs and demonstrated their association with poor prognosis in BC [11,36]. Several studies also stated that high levels of CD56+ NK cells were significantly related to better outcomes in solid tumors. Moreover, the decrease in NK cells infiltration might predict failure of response to chemotherapy in BC [37,38]. CD8+ CTLs in BC were investigated by Oshi et al. [39] who revealed that a high CD8 score was significantly related to a good prognosis and better survival.

In addition to the role of MACC1 in cancer metastasis and progression, MACC1 was suggested to have a crucial role in the TME and the immune escape mechanisms [40]. To our knowledge, this study is the first to investigate the association between MACC1 mRNA relative expression and immune cells in the TME of BC. In this study, we analyzed the correlation between MACC1 mRNA relative expression and the different immune cells in BC TME (CD163+ TAMs, CD56+ NK cells, and CD8+ CTLs).

The present study reported a significant positive correlation between MACC1 mRNA relative expression and CD163+ TAMs counts, whereas the increase in MACC1 mRNA relative expression levels was significantly correlated with a decrease in CD56+ NK cells and CD8+ CTLs counts.

MACC1 could prevent immune attacks via stimulation of TIMP1/2, which proactively recruits TAMs [41]. TAMs initiate a tumor-promoting microenvironment and may cause tumor evasion from immune destruction by elaborating various cytokines (e.g., IL1, IL6, TNF-α) and growth factors (e.g., TGF-β) that reduce T-cell activity and T cell-mediated tumor elimination [42].

Moreover, MACC1 could also facilitate immune escape of tumors as it downregulates the expression of activated receptor NKG2D in NK cells by up-regulating TGF-β1, thus inhibiting the killing activity of NK cells, and facilitating tumor immune evasion [40]. Previous studies postulated that MACC1 controls PDL1 expression and tumor immunity by modulation of the c-Met/AKT/mTOR pathway, hence affecting cytotoxic T-cells in BC TME [43]. PD-1/PD-L1 interaction might promote tumor escape, as it induces apoptosis of activated T-cells, thereby exerting a negative role in tumor immunity [44].

Analysis of MACC1 mRNA relative expression and the patients’ survival revealed that high MACC1 expression was significantly associated with poor OS and PFS. Additionally, multivariate Cox regression analysis demonstrated that MACC1 could serve as an independent prognostic factor in BC. These findings were similar to Tan et al. [29], who reported that high serum MACC1 level was correlated with poor disease-free survival and could be an independent prognostic factor for BC. Moreover, Huang et al. [31] found that high MACC1 expression was significantly associated with reduced OS and PFS in BC patients.

It is noteworthy that there are some limitations in this study, as the association of MACC1 expression with other more relevant immune suppression markers, such as PD1 and PD-L1, was not investigated. It is also difficult to adopt the results of MACC1 expression in breast cancer subtypes, particularly TNBC, given the relatively small number of TNBC cases in this study. Therefore, further studies are recommended to include immune checkpoint markers and focus on the relationship between MACC1 expression and breast cancer subtypes. In addition, concordance analysis between relative MACC1 mRNA expression and IHC expression should be taken into account.

## 5. Conclusions

This study identified a significant association between MACC1 expression and BC prognosis. MACC1 was highly expressed in BC tissues and high MACC1 expression was significantly associated with poor prognostic factors and poor OS and PFS. MACC1 could act as an independent prognostic factor for BC. High MACC1 expression was significantly correlated with an increase in CD163+ TAMs and a decrease in both CD56+ NK cells and CD8+ CTLs. Thus, MACC1 might affect immune cells infiltration in the TME and enhance tumor cells immune escape.

## Figures and Tables

**Table 1:** Patients’ characteristics.

	No. = 120 (%)
Age (Years)	
Mean ± SD.	47.3 ± 5
Median (Min.–Max.)	48 (36–56)
Menopausal status	
Premenopausal	52 (43.3%)
Postmenopausal	68 (56.7%)
Tumor size (cm)	
≤2	38 (31.7%)
>2–≤5	60 (50%)
>5	22 (18.3%)
Histologic grade	
Grade II	88 (73.3%)
Grade III	32 (26.7%)
Nodal involvement	
Absent	16 (13.3%)
Present	104 (86.7%)
Lymphovascular invasion	
Absent	103 (85.8%)
Present	17 (14.2%)
Tumor stage	
I	35 (29.2%)
II	21 (17.5%)
III	64 (53.3%)
ER	
Negative	48 (40%)
Positive	72 (60%)
PR	
Negative	56 (46.7%)
Positive	64 (53.3%)
HER2 receptor	
Negative	78 (65%)
Positive	42 (35%)
Ki-67	
<15	50 (41.7%)
≥15	70 (58.3%)
Molecular subtypes	
Luminal A	36 (30%)
Luminal B	65 (54.2%)
HER2 enriched	8 (6.7%)
TNBC	11 (9.1%)
CD163+ TAMs count	
Mean ± SD.	31.6 ± 14.8
Median (Min.–Max.)	34.5 (0–53)
CD56+ NK cells count	
Mean ± SD.	30.8 ± 18.1
Median (Min.–Max.)	29 (0–60)
CD8+ CTLs count	
Mean ± SD.	52.2 ± 23.4
Median (Min.–Max.)	47.5 (13–95)
Survival (3 years)	
Dead	16 (13.3%)
Alive	104 (86.7%)
Recurrence	
No	84 (70%)
Yes	36 (30%)

SD: standard deviation, ER: Estrogen receptor, PR: Progesterone receptor, HER2: Human epidermal factor receptor 2, TNBC: Triple negative breast cancer, TAMs: Tumor-associated macrophages, NK cells: natural killer cells, CTLs: cytotoxic T lymphocytes.

**Table 2:** Relationship between MACC1 expression and clinicopathological parameters.

		MACC1 Expression		
	*n* = 120	Median	Range	*p*-Value
Menopausal status				
Premenopausal	52	1.8	0.5–3.2	0.567
Postmenopausal	68	1.8	0.4–3.1	
Tumor size (cm)				
≤2	38	1.3	0.5–3.0	<0.001 *
>2–≤5	60	1.8	0.4–3.1	
>5	22	2.5	1.3–3.2	
Histologic grade				
Grade II	88	1.4	0.4–3.0	<0.001 *
Grade III	32	2.3	1.2–3.2	
Nodal involvement				
Absent	16	0.7	0.5–2.1	0.001 *
Present	104	2.0	0.4–3.2	
Lymphovascular invasion				
Absent	103	1.5	0.4–3.2	<0.001 *
Present	17	2.5	1.6–3.2	
Tumor stage				
I	35	0.9	0.5–3.0	<0.001 *
II	21	1.2	0.4–2.4	
III	64	2.3	0.5–3.2	
ER				
Negative	48	2.10	0.50–3.20	0.106
Positive	72	1.50	0.40–3.10	
PR				
Negative	56	1.95	0.50–3.20	0.091
Positive	64	1.65	0.4–3.00	
HER_2_ receptor				
Negative	78	1.75	0.40–3.00	0.061
Positive	42	1.90	0.50–3.20	
Ki-67				
<15	50	0.7	0.4–3.0	<0.001 *
≥15	70	2.3	0.5–3.2	
Molecular subtypes				
Luminal A	36	1.14	0.4–3.0	
Luminal B	65	2.06	0.5–3.2	<0.001 *
HER2 enriched	8	1.74	0.5–3.2	
TNBC	11	1.9	0.5–2.9	

* Statistically significant (*p* ≤ 0.05), ER: Estrogen receptor, PR: Progesterone receptor, HER2: Human epidermal factor receptor 2, TNBC: Triple negative breast cancer.

**Table 3:** Univariate and multivariate COX regression analysis of prognostic markers for mortality.

	Univariate		Multivariate ^#^	
	*p*-Value	HR (95% C.I)	*p*-Value	HR (95% C.I)
Tumor size (>5 cm)	0.456	1538 (0.496–4769)		
Nodal involvement	0.300	25.402 (0.056–11557.02)		
Lymphovascular invasion	<0.001 *	7122 (2665–19.031)	0.042 *	3138 (1040–9471)
Stage (III)	0.042 *	64.419 (1154–3597.441)	0.943	54.893.102 (0.0–1004)
ER	0.409	0.662 (0.248–1763)		
PR	0.027 *	3574 (1.152–11.084)	0.406	0.560 (0.142–1128)
HER2 receptor	0.889	1.075 (0.391–2958)		
Ki-67	0.178	2176 (0.70–6748)		
CD163	0.001 *	1106 (1042–1173)	0.083	1058 (0.993–1128)
CD56	0.001 *	0.942 (0.909–0.976)	0.207	0.979 (0.947–1012)
CD8	0.267	0.988 (0.966–1010)		
MACC1 expression	<0.001 *	12.690 (3510–45.876)	0.015 *	5932 (1423–24.738)

* Statistically significant (*p* ≤ 0.05); #: All variables with *p* < 0.05 were included in the multivariate; ER: Estrogen receptor, PR: Progesterone receptor, HR: Hazard ratio, C.I: Confidence interval.

## Data Availability

Data used and/or analyzed during this study are not available for public access because of patient privacy concerns but are available from the corresponding author upon reasonable request.

## Abbreviations

BC	Breast cancer;
CTLs	Cytotoxic T lymphocytes;
DAB	diaminobenzidine;
ER	Estrogen receptor
HER2	Human epidermal receptor 2;
HGF	Hepatocyte growth factor;
HRP	Horseradish peroxidase;
LHRH	Luteinizing-hormone-releasing hormone;
LVI	Lymphovascular invasion;
MACC1	Metastasis-associated colon cancer-1;
NK cells	Natural killer cells;
OS	Overall survival;
PCR	Polymerase chain reaction;
PFS	Progression-free survival;
PR	Progesterone receptor;
TAMs	Tumor-associated macrophage;
TIIC	Tumor-infiltrating immune cells;
TME	Tumor microenvironment;
WHO	World Health Organization.
